# A fluorine-absorbing and mechanically elastic binder with triangular architecture enables both bulk- and interface-stable Si anodes

**DOI:** 10.1039/d5sc09750a

**Published:** 2026-01-26

**Authors:** Zhipeng Wang, Qitao Shi, Weiqi Song, Luwen Li, Jiaqi Wang, Cheng Zhang, Alicja Bachmatiuk, Chen Lu, Peichao Zou, Jinho Choi, Yanbin Shen, Ruizhi Yang, Mark H. Rümmeli

**Affiliations:** a Soochow Institute for Energy and Materials Innovation, College of Energy, Key Laboratory of Advanced Carbon Materials and Wearable Energy Technologies of Jiangsu Province, Key Laboratory of Core Technology of High Specific Energy Battery and Key Materials for Petroleum and Chemical Industry, Soochow University Suzhou 215006 China mhr1967@yahoo.com mhr1@vsb.cz; b i-Lab, CAS Center for Excellence in Nanoscience, Suzhou Institute of Nano-Tech and Nano-Bionics (SINANO), Chinese Academy of Sciences (CAS) Suzhou 215123 P. R. China; c School of Electronic and Information Engineering, Changshu Institute of Technology Changshu 215500 China; d Faculty of Chemistry, Wrocław University of Science and Technology Wybrzeże Wyspiańskiego 27 50-370 Wrocław Poland; e Electron Beam Emergent Additive Manufacturing (EBEAM) Center, Center for Nanotechnology (CNT), Center for Energy and Environmental Technologies (CEET), VSB—Technical University of Ostrava 17. Listopadu 15 70833 Ostrava Czech Republic; f Institute for Materials Chemistry, IFW Dresden 20 Helmholtz Strasse 01069 Dresden Germany

## Abstract

Mechanically robust polyacrylic acid (PAA) binders are extensively investigated for improving the structural stability and extending the cycle life of Si anodes. However, PAA cannot simultaneously suppress interfacial side reactions, preserve structural integrity, and ensure efficient ion transport. This paper presents a mechanically elastic polymeric binder, PCZn, that integrates locally positive charges to introduce a LiF-rich interface and high ionic conductivity within a triangular architecture established through the triadic interaction of a long-chain PAA adhesive, cross-linking agent chitosan oligosaccharide, and cation donor zinc gluconate. PCZn imparts a highly reversible anti-strain capability, a conformal LiF-rich solid-electrolyte-interface layer, and high ionic conductivity to Si anodes, resulting in remarkable electrochemical performance with a high capacity of 1210 mAh g^−1^ after 450 cycles at 3 A g^−1^ and enhanced fast-charging capability of 1468 mAh g^−1^ at 8 A g^−1^. Thus, concurrently addressing mechanical failure, interfacial instability, and sluggish kinetics of Si anodes through advanced binder design will help develop high-energy-density next-generation batteries with long cycle lives.

## Abstract 2

Mechanically robust polyacrylic acid (PAA) binders are extensively investigated for improving the structural stability and extending the cycle life of Si anodes.

## Graphical abstract

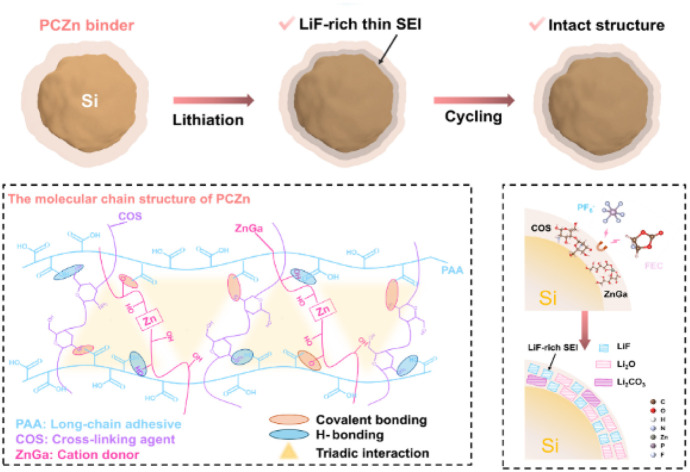

## Introduction

Lithium-ion batteries (LIBs), which are valued for their long cycle lifetimes and low cost, are widely utilized in portable electronics and electric vehicles.^1^ However, the inherent energy-density limitations of commercial graphite anodes impede their ability to meet the escalating demand for lightweight, high-capacity energy storage.^2^ Owing to its ultrahigh theoretical capacity (4200 mAh g^−1^), low cost, natural abundance, and environmental friendliness, Si has emerged as a highly promising next-generation anode material.^3^ However, Si undergoes substantial volume expansion (∼300%) during lithiation–delithiation processes, which creates critical problems including particle pulverization, electrode delamination, and the formation of an unstable solid electrolyte interface (SEI).^4^ Solutions to these issues have been explored extensively; key strategies include the use of Si nanostructuring, morphological engineering, and protective interfacial coatings, along with the design of advanced binders.^5–9^ Among these solutions, the application of rationally designed binders is becoming increasingly pivotal, enabling significant enhancements in the electrochemical performance of Si anodes.

Aqueous polymers, such as carboxymethyl cellulose, polyvinyl alcohol, and polyacrylic acid (PAA), are regarded as advanced binders for Si anodes because of their ability to form hydrogen bonds.^10^ In particular, PAA has been researched extensively because of its abundant carboxyl groups, which can form strong hydrogen bonds with Si particles to mitigate their substantial volume changes.^11^ However, the PAA binder intrinsically suffers from high rigidity owing to the over-crosslinking of PAA chains by the hydrogen bonds, and this rigidity directly induces irreversible mechanical failure in the associated electrodes during cycling.^12^ Current strategies to improve the properties of PAA include the introduction of molecules that can form covalent bonds or hydrogen bonds with carboxyl groups^9,11,13,14^ to enhance the mechanical strength of the adhesive network. Additionally, the roles beyond mechanical bonding cannot be neglected, because the binder also acts as an artificial layer on the Si particles.^15^ An ideal binder for Si-based anodes should integrate the following characteristics: (1) high elasticity and strong adhesion to limit electrode expansion while maintaining intimate contact with the current collector, (2) high ionic conductivity to improve the rate capability of the Si anodes, and (3) the ability to induce the formation of a robust, LiF-rich SEI for sustained interfacial stability. At present, binders that simultaneously satisfy these requirements are rare.

**Fig. 1:**
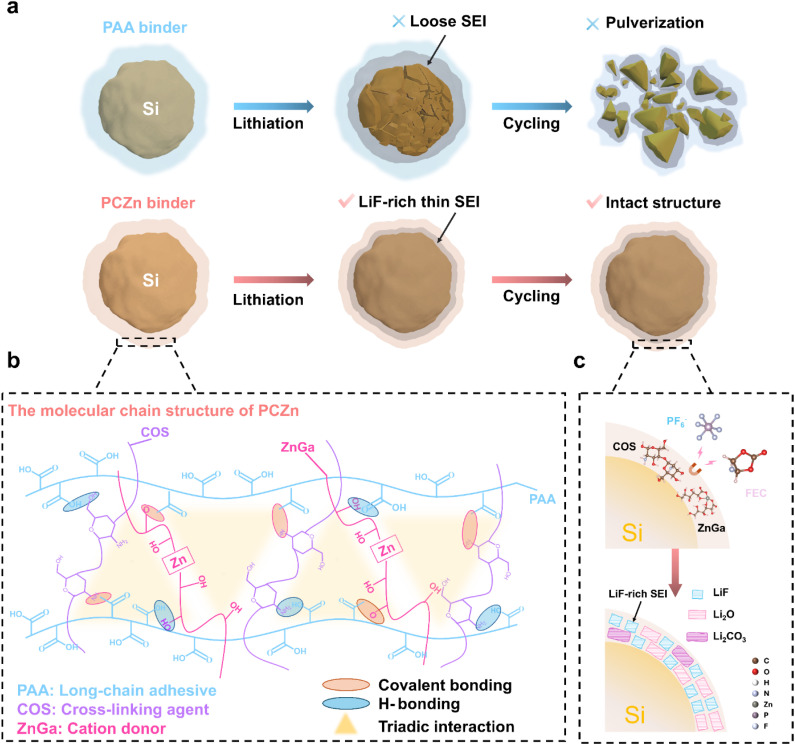
(a) Schematic illustrations showing the operation of Si electrodes with different binders during cycling. (b) Molecular structure and interactions among chains of PCZn binder. (c) Illustration of the formation process of LiF-rich SEI derived from the interaction between PCZn binder and electrolyte.

In this paper, we report a multifunctional polymeric binder, PCZn, that synergistically integrates interfacial bonding capability, a mechanically elastic framework, F-absorbing sites, and a highly conductive Li-ion pathway within a triangular architecture. As a result, the PCZn binder stabilizes both the bulk phase and the interface layer of the Si anodes under repeated stress (Fig. 1a). The PCZn binder is established through a triadic interaction among a long-chain PAA adhesive, chitosan oligosaccharide (COS), a low-cost, water-soluble crosslinking agent, and zinc gluconate (ZnGa), a cation donor (Fig. 1b). PAA serves as the long-chain polymeric backbone, providing robust foundational adhesion and processability. COS acts as a multi-functional crosslinking agent and modulator. Its amino groups are crucial for guiding interfacial chemistry, working in tandem with ZnGA to promote the formation of a superior, LiF-rich SEI, as confirmed by XPS and TEM. ZnGa functions as a dynamic crosslinker and cation donor, enabling efficient stress dissipation and enhancing ionic transport within the network. Fig. 1c schematically illustrates the LiF-rich SEI formation mechanism, where formation occurs through synergistic interactions between the COS/ZnGa components in the PCZn binder and the electrolyte. Hence, the as-prepared Si electrode achieved a high capacity of 1210 mAh g^−1^ after 450 cycles at 3 A g^−1^. Additionally, PCZn exhibited high ionic conductivity, enabling excellent rate capability with a capacity of 1468 mAh g^−1^ at 8 A g^−1^. This work elucidates the potential of binders for maintaining both the bulk and interface stabilities of Si-based anodes.

## Results and discussion

**Fig. 2:**
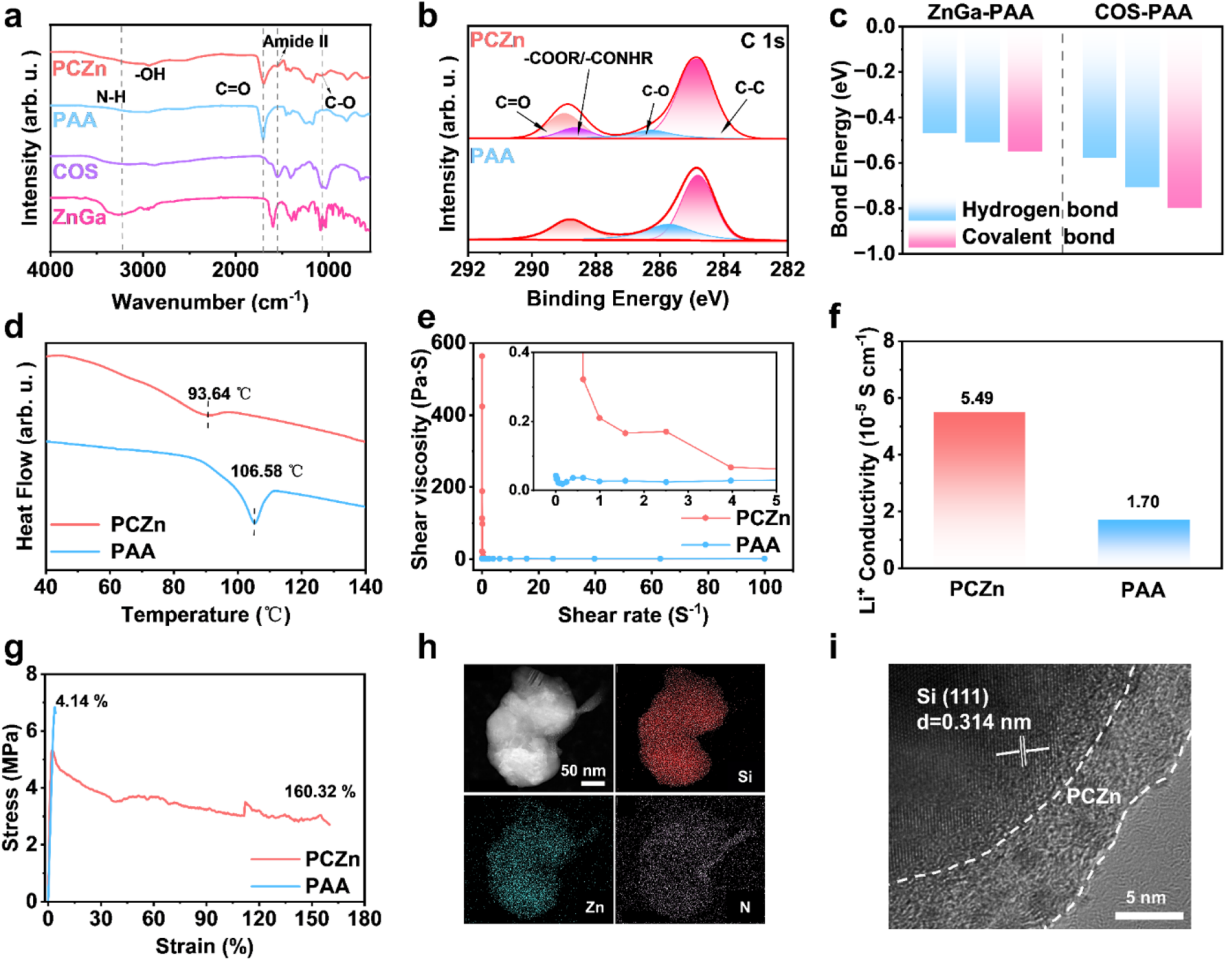
(a) FTIR spectra of PCZn, PAA, COS, and ZnGa. (b) C 1s high-resolution XPS spectra of PCZn and PAA. (c) Calculated energies of gradient hydrogen bonds and covalent bonds. (d) DSC curves of PCZn and PAA. (e) Shear viscosities of PCZn and PAA solutions (2 wt%). (f) Li^+^ conductivity of PCZn and PAA. (g) Stress–strain curves of PCZn and PAA. (h) HADDF and EDS mappings of Si–PCZn. (i) High-resolution TEM image of Si–PCZn.

The synthesized PCZn binder was first characterized using FTIR spectroscopy, which revealed a redshift of the C=O stretching peak from 1702 cm^−1^ in the PAA spectrum to 1694 cm^−1^ in the PCZn spectrum (Fig. 2a), indicating the formation of hydrogen bonds among COS, ZnGa, and PAA.^16^ A distinct peak at 1551 cm^−1^, characteristic of amide II (coupled N–H bending and C–N stretching), was observed in the PCZn spectrum only,^17^ confirming the formation of amide groups (–CONHR) in PCZn. The peak observed at 1064 cm^−1^, corresponding to C–O stretching, suggested the presence of ester groups (–COOR)^18^ and revealed the formation of covalent bonding within PCZn. To further verify the presence of amides and esters, X-ray photoelectron spectroscopy (XPS) analysis was performed. An additional peak was observed at 288.6 eV in the C 1s spectrum of PCZn, corresponding to the amide/ester carbon species (Fig. 2b).^19^ A peak at 400.84 eV appeared in the N 1s spectrum, confirming the presence of amide nitrogen.^20^ The O 1s spectrum featured a peak at 532.5 eV, associated with the ether oxygen (–O–) in aliphatic esters (Fig. S1),^21^ further confirming the presence of ester groups in the binder. The calculated energy of gradient hydrogen bonds and covalent bonds shown in Fig. 2c, S2, and S3 indicate the thermodynamic feasibility of the PCZn formation.

The entanglement of molecular chains would cause the failure of PAA binders. In contrast, some carboxyl groups along PAA chains are bonded to other components in the PCZn binder and lead to lower zeta potential (Fig. S4).^22^ Differential scanning calorimetry (DSC) results showed that the glass transition temperature (*T*_g_) of PCZn was significantly lower than that of PAA (Fig. 2d), because the bonding between COS/ZnGa and the PAA carboxyl groups reduced the intermolecular chain interactions and increased chain mobility.^23^ Furthermore, rheological measurements confirmed that a three-dimensional network structure formed by the bonding of COS and ZnGa with PAA substantially enhanced the overall binder viscosity (Fig. 2e).^24^ The Li^+^ conductivities of the two binders were measured using electrochemical impedance spectroscopy (EIS) using a symmetric cell with ionically blocking electrodes (Fig. S5). The ionic conductivity of PCZn binder was approximately 2.2 times higher than that of PAA binder (Fig. 2f). Note that the –NH groups in the PCZn binder inhibit anion migration, whereas the hydrogen bonding and steric hindrance of the polymer backbone impede solvent access to Li^+^. These combined effects facilitate Li^+^ desolvation and promote the formation of efficient Li^+^ transport channels. We conducted two key complementary experiments: Li^+^ transference number measurement and electrolyte swelling tests The elevated Li^+^ transference number (*t*Li^+^ = 0.64) substantiates the role of –NH groups in suppressing anion migration. The lower electrolyte swelling ratio validates the role of the dense, hydrogen-bonded network in impeding solvent molecule access (Fig. S6 and S7).^25^

The mechanical properties of the binders were evaluated by tensile testing (Fig. 2g). The PCZn film exhibited exceptional ductility, with a strain at break of 160.32%, compared with 4.14% for PAA. This substantially higher strain indicates superior elasticity and the ability to withstand significant external stress without fracture. To demonstrate the uniform coating of Si particles by PCZn, energy-dispersive spectroscopy (EDS) mapping of transmission electron microscopy (TEM) images was performed (Fig. 2h). Uniform distributions of Zn (from ZnGa) and N (from COS) on the Si surface were observed, indicating the formation of a uniform PCZn polymer network on Si. Furthermore, high-resolution TEM (HRTEM) imaging revealed a PCZn coating layer approximately 6 nm thick that conformally covered the Si particles, serving as an artificial interface (Fig. 2i).

**Fig. 3:**
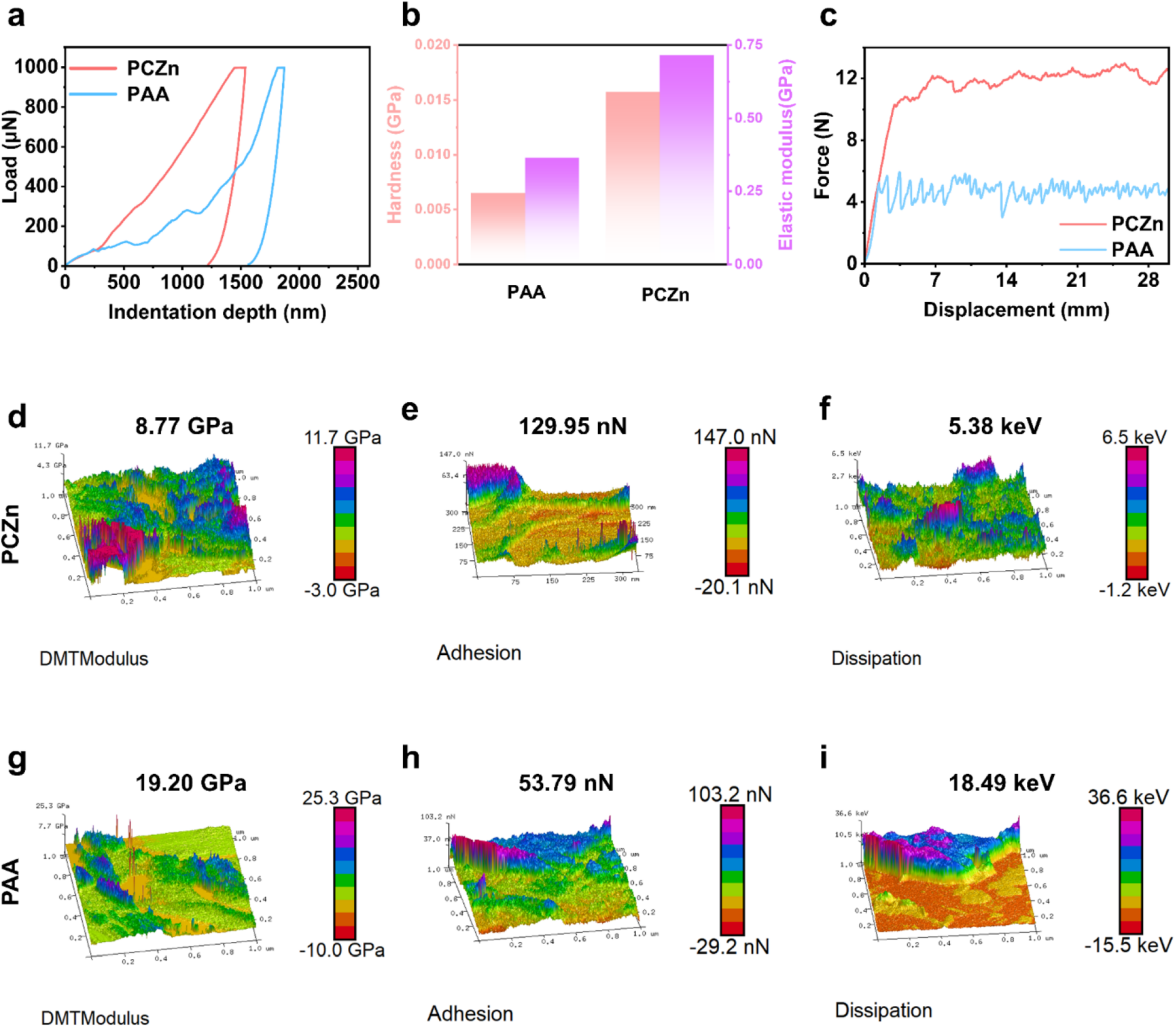
(a) Load-indentation depth curves, (b) hardness and modulus values determined using nano-indentation tests, (c) peeling test results, and (d)–(i) DMT modulus, adhesion, and dissipation mapping results for Si–PCZn and Si–PAA electrodes.

The mechanical strength of the Si electrode prepared with the PCZn binder (hereafter referred to as the “Si–PCZn electrode”) and that of the Si–PAA electrode were measured by nano-indentation, a 180° peeling strength test, and atomic force microscopy (AFM). As shown in Fig. 3a, when an identical force was loaded onto the electrodes, a lower indentation depth was observed for the Si–PCZn electrode, corresponding to a significantly higher hardness and elastic modulus than those of the Si–PAA electrode (Fig. 3b).^26^ The 180° peel strength test results showed that PCZn exhibited adhesion strength that was two times greater than that of PAA (Fig. 3c); this property enabled tight bonding of the Si–PCZn electrode materials to the Cu foil.

AFM measurements revealed a lower modulus for the PCZn electrode compared to that of the PAA electrode (Fig. 3d, g and S8), indicating that the incorporation of small molecules enhances binder toughness.^27^ Additionally, AFM adhesion force mapping revealed significantly stronger adhesion for PCZn (91.25 nN) compared to that for PAA (53.79 nN). This enhanced adhesion, which was consistent with the 180° peel test results, suggested improved binder connection to the current collector. As a result, active-material detachment and subsequent electrochemical isolation were mitigated (Fig. 3e, h and S9). Furthermore, lower energy dissipation (5.34 keV) was observed for the Si–PCZn electrode composite compared to that for the Si–PAA electrode (Fig. 3f, i and S10). This result demonstrated that the energy caused by Si deformation could be dissipated over time through dissociation of the hydrogen bonds in the PCZn binder. Additionally, this finding indicated reduced irreversible deformation and microcrack formation within the PCZn binder under applied stress.^28,29^

A uniformly coated PCZn binder acts as an interface between the Si particles and electrolytes. To confirm the role of the PCZn coating, we conducted FTIR and Raman tests on the electrolyte with and without added PCZn. The FTIR results indicated lower C–F intensity in the electrolyte with added PCZn, suggesting that the C–F bond in the FEC formed a coordination with the PCZn. This result indicates from the side that ZnGa has an adsorption effect on FEC (Fig. S11), a finding that is supported by the small peak that emerged after 1080 cm^−1^ in the Raman spectrum (Fig. S12).^30^ Contact-angle tests were employed to measure the interfacial wettability of the PCZn electrode exposed to the electrolyte. A smaller contact angle was observed for the PCZn electrode compared to that for the PAA electrode (Fig. S13), indicating enhanced electrolyte penetration, which facilitated Li^+^ transportation through the interface.^31^

**Fig. 4:**
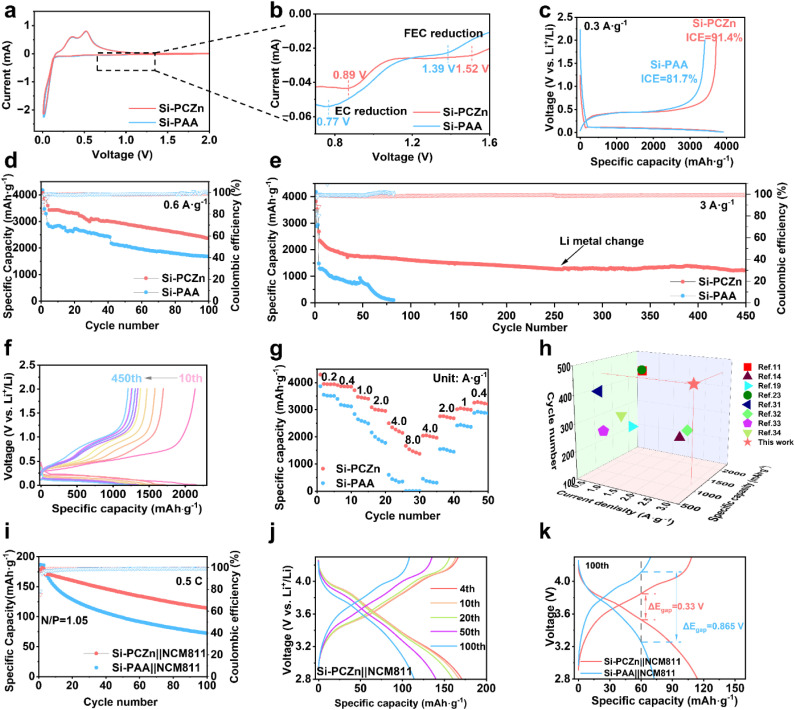
(a) CV curves of Si–PCZn and Si–PAA electrodes and (b) enlarged curves for the first cycle. (c) Charge–discharge curves of Si–PCZn and Si–PAA electrodes at a current density of 0.3 A g^−1^. Cycling performance of Si–PCZn and Si–PAA electrodes at current densities of (d) 0.6 and (e) 3 A g^−1^. (f) Charge–discharge curves of the Si–PCZn electrode at a current density of 3 A g^−1^. (g) Rate performance of Si–PCZn and Si–PAA electrodes. (h) Comparison of the electrochemical performance of the Si–PCZn anode with previous reports for Si-based anodes. (i) Cycling performance of Si–PCZn‖NCM811 and Si–PAA‖NCM811full cells at 0.5C. (j) Charge–discharge profiles for the fourth, tenth, 20th, 50th and 100th cycles of the Si–PCZn‖NCM811 full cell. (k) Voltage polarization curves of Si–PCZn‖NCM811and Si–PAA‖NCM811 full cells.

The electrochemical properties of the Si–PCZn and Si–PAA electrodes were evaluated using various electrochemical methods. Cyclic voltammetry (CV) curves for the initial cycles of both electrodes revealed similar electrochemical behaviors (Fig. 4a). Notably, for the Si–PAA electrode, the peaks associated with FEC and ethylene carbonate (EC) decomposition in the electrolyte were 1.39 and 0.77 V, respectively; however, when PCZn was employed as a binder, the decomposition peaks of the FEC and EC in the electrolyte shifted to higher potentials of 1.52 and 0.89 V, respectively (Fig. 4b). The advanced reduction and enhanced peak intensity of the FEC decomposition indicate favorable kinetics and a lower energy barrier on the electrode surface, which facilitated the formation of a dense, LiF-rich SEI. The underlying reason for this behavior is likely the adsorption of the FEC by the ZnGa component of the PCZn binder, which caused FEC enrichment on the Si particle surfaces and, thus, promoted its decomposition. This finding is consistent with the results of the Raman and FTIR analyses reported in the previous section for electrolytes with and without PCZn.^32^ Furthermore, the early appearance of the EC decomposition peak at the Si–PCZn electrode indicates that the use of the PCZn binder significantly accelerates the kinetic process of the reduction reaction. Fig. 4c shows the charge–discharge profiles of both electrodes for the first cycle at a current density of 0.3 A g^−1^. A slightly lower discharge capacity but a higher charge capacity was observed for the Si–PCZn electrode, yielding an ICE value of 91.35%, which was markedly superior to the 81.73% ICE value obtained for the Si–PAA electrode. The superior ICE value indicates that the PCZn binder effectively mitigated irreversible side reactions at the electrode–electrolyte interface, thereby reducing the irreversible capacity loss. Furthermore, analysis of the coulombic efficiency (CE) over the first ten cycles (Fig. S14) revealed that the Si–PCZn electrode exceeded 99% CE after only five cycles and exhibited excellent reversibility during the lithiation–delithiation processes.

The long-term cycling stability of the electrodes was evaluated through charge–discharge tests at a current density of 0.6 A g^−1^. The Si–PCZn electrode maintained a capacity of 2374 mAh g^−1^ after 100 cycles, far exceeding the value of 1685 mAh g^−1^ obtained for the Si–PAA electrode (Fig. 4d). At a raised current density of 3 A g^−1^, accelerated capacity fading and fluctuating CE were observed beyond 50 cycles for the Si–PAA electrode; these behaviors were due to Si particle pulverization and repetitive SEI formation.^33^ After 85 cycles, the Si–PAA electrode lost its electrochemical activity. In sharp contrast, the Si–PCZn electrode retained an excellent capacity of 1210 mAh g^−1^ after 450 cycles, along with lower voltage polarization (Fig. 4e and f). As shown in Fig. S15, PAA systems with individually added single components of COS or ZnGa exhibited significantly inferior cycling performance compared to that of the PCZn system incorporating both COS and ZnGa. These results indicate a synergistic interaction between the two components, which yielded enhanced cycling stability of the Si anode. Rate capability tests demonstrated the fast-charging capability of the Si–PCZn electrode (Fig. 4g). That is, the Si–PCZn electrode delivered reversible capacities of 3950, 3842, 3400, 2951, 2143, and 1468 mAh g^−1^ at current densities of 0.2, 0.4, 1, 2, 4, and 8 A g^−1^, respectively, whereas the capacity of the Si–PAA electrode diminished to near zero at 8 A g^−1^. We conducted a systematic electrochemical impedance spectroscopy (EIS) study on both the Si–PCZn electrode and a reference Si–PAA electrode after prolonged cycling (100 cycles) at different rates (1 A g^−1^ and 8 A g^−1^). After the harsh 8 A g^−1^ cycling, the Si–PCZn electrode exhibits remarkably stable interface resistances. Notably, its *R*_ct_ shows a slight decrease, and the *R*_sei_ increases only minimally. In stark contrast, the Si–PAA electrode suffers from drastic interfacial degradation after 8 A g^−1^ cycling. Both *R*_sei_ and *R*_ct_ increase dramatically by approximately 106% and 158%, respectively. (Fig. S16) When the current density recovered to 0.4 A g^−1^, the Si–PCZn electrode achieved a capacity of 3281 mAh g^−1^, demonstrating outstanding electrochemical reversibility and structural stability. A comparative analysis with previously reported Si-based anodes (Fig. 4f and Table S1) established the competitive advantage of our Si–PCZn electrode in terms of cycling performance, thereby validating the effectiveness of our binder design strategy. The radar chart clearly demonstrates that the PCZn binder achieves superior or highly competitive performance in four of the five key metrics. Therefore, the novelty of the PCZn binder lies in its synergistic integration of multiple, critical functions into a single, coherent “triangular architecture.” Unlike many advanced binders that excel primarily in one domain (*e.g.*, conductivity or mechanics or SEI modulation), our design simultaneously and effectively addresses the core trilemma (Fig. S17).^11,14,19,23,34–37^

To further evaluate the practicality of the Si–PCZn anode when employed in LIBs, the electrode was paired with an NCM811 cathode to construct a full battery with N/P = 1.05. The Si–PCZn‖NCM811 full cell exhibited an initial reversible capacity of 178 mAh g^−1^ within the voltage range of 2.8–4.3 V. Compared with Si–PAA‖NCM811, the Si–PCZn‖NCM811 cell delivered a more stable cycle performance. After 100 cycles at 0.1 A g^−1^ (0.5C), the Si–PCZn‖NCM811 cell maintained a capacity of 115 mAh g^−1^, whereas the capacity of the Si–PAA‖NCM811 cell was only 72 mAh g^−1^, at a current density of 0.2 A g^−1^ (1C), the high capacity retention rate advantage of Si–PCZn becomes even more pronounced. (Fig. 4i, j and S18). The voltage polarizations (Δ*E*_p_) of both cells measured after 100 cycles are shown in Fig. 4k. The Si–PCZn‖NCM811 full cell exhibited a Δ*E*_p_ of 0.33 V, which was significantly lower than that of Si–PAA‖NCM811 (0.865 V), indicating fast ion transportation along the PCZn binder.

**Fig. 5:**
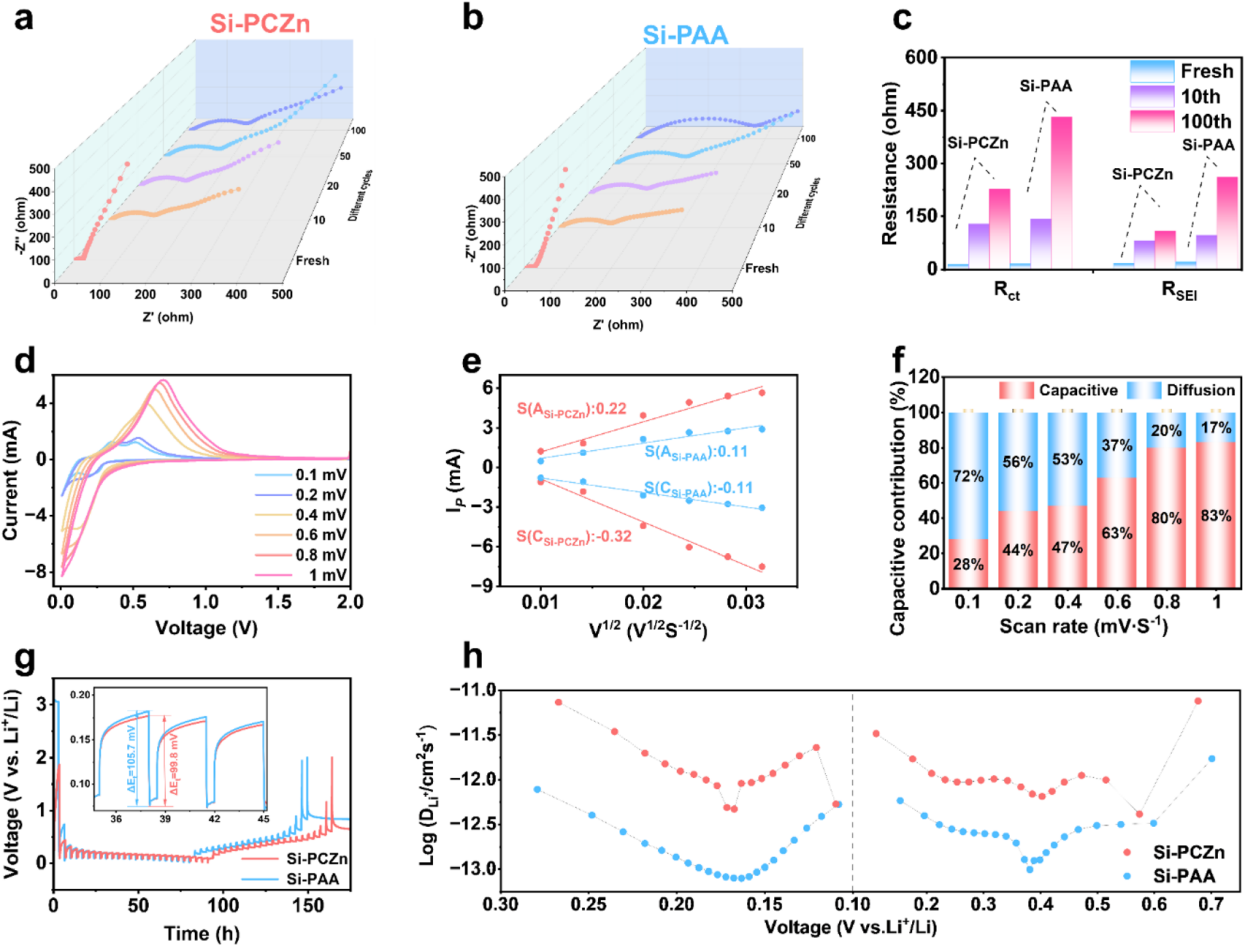
(a)–(c) EIS spectra and fitted results for the Si–PCZn and Si–PAA electrodes after different numbers of cycles. (d) CV curves of Si–PCZn under different sweep rates from 0.1 to 1 mV s^−1^. (e) Plots of CV peak current *versus* the square root of the scanning rates. (f) Capacitance contributions of the Si–PCZn electrode at various sweep rates. (g) GITT test results for the Si–PCZn and Si–PAA electrodes. (h) Li-ion diffusion rates calculated for the Si–PCZn and Si–PAA electrodes.

EIS was used to probe the origin of the superior electrochemical performance of the Si–PCZn electrode. As shown by the spectra in Fig. 5a and b, both the interfacial resistance (*R*_SEI_) and charge-transfer resistance (*R*_ct_) of the Si–PCZn and Si–PAA electrodes increased upon cycling; however, the increases for the Si–PCZn electrode were markedly smaller than those for the Si–PAA electrode. Nyquist plots for both electrodes were fitted with an equivalent-circuit model (Fig. S19);^38^ the fitted values are summarized in Fig. 5c. For the Si–PCZn electrode, *R*_ct_ was 127.2 and 227 Ω after the first cycle and after 100 cycles, respectively. These values were substantially lower than the corresponding values of 140.8 and 432 Ω for the Si–PAA electrode. Moreover, the change in the SEI resistance (*R*_SEI_) from the first cycle to 100 cycles was much smaller for the Si–PCZn electrode than for the Si–PAA electrode. The markedly lower *R*_ct_ and *R*_SEI_ are attributed to the enhanced ionic conductivity of the PCZn binder, together with the high elastic buffering capability and a LiF-rich SEI. CV test results obtained at different scanning rates are shown in Fig. 5d and S20. The CV peak currents of the respective redox peaks were plotted against the square roots of the scanning rates for both electrodes (Fig. 5e). The slope of the fitting curve obtained for the Si–PCZn electrode exceeded that obtained for the Si–PAA.^39^ This result indicates that the Si–PCZn electrode had faster Li^+^ ion diffusion kinetics. The diffusion-control and capacitance-behavior contributions were calculated by fitting the CV curve. The overall contribution was calculated as

1 *i*(*V*) = *K*_1_*V* + *K*_2_*V*^1/2^

where *K*_1_*V* and *K*_2_*V*^1/2^ represent the diffusion-control and capacitance-behavior contributions, respectively. As shown in Fig. 5f, S21 and S22, for both Si–PCZn and Si–PAA, the capacitive contribution ratio increased with the scan rate. Notably, at a 1 mV s^−1^ scan rate, the capacitive contribution reached 83% for Si–PCZn, which was substantially higher than the 77% observed for Si–PAA. This result indicates that the Si–PAA charge storage process was more significantly limited by the ion diffusion kinetics, a finding that is consistent with its slower interfacial kinetics.^40^

To evaluate the Li-ion diffusion coefficient (*D*_Li_^+^), galvanostatic intermittent titration technique (GITT) measurements were performed. The voltage–time curves of the two electrodes are shown in Fig. 5g. A lower voltage drop was observed for Si–PCZn than for Si–PAA, indicating that the Si–PCZn electrode had faster ion transport kinetics and a shorter diffusion path. The calculated Li-ion diffusion rate also supports this finding (Fig. 5h).^41^

**Fig. 6:**
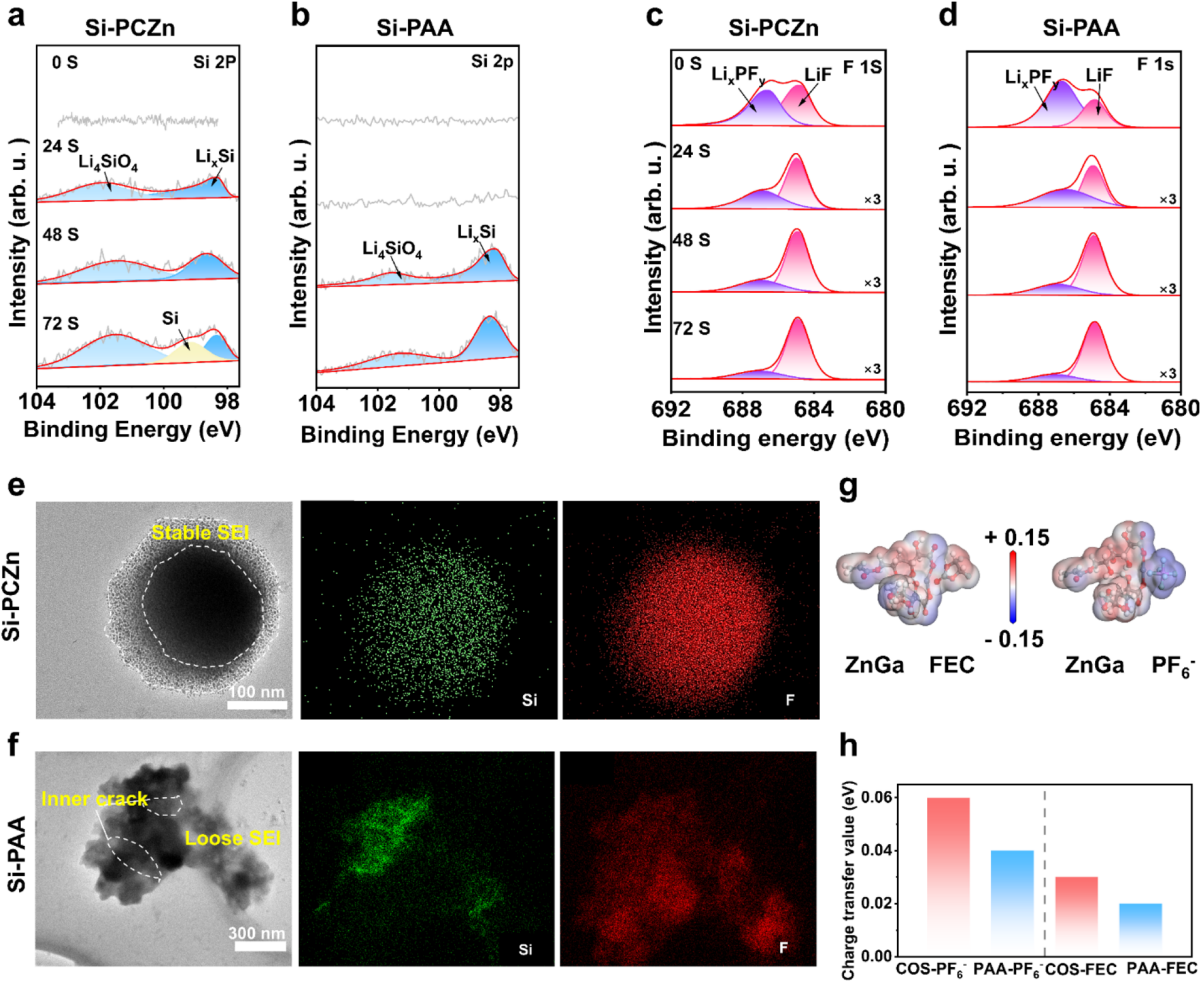
High-resolution XPS spectra of (a) and (b) Si 2p and (c) and (d) F 1s from cycled Si electrodes. TEM image and corresponding EDS mapping of (e) Si–PCZn and (f) Si–PAA electrodes after 50 cycles. (g) Dipole–dipole interaction between ZnGa and FEC and ZnGa and PF_6_^−^. (h) Charge density differences between COS and PAA.

To characterize the unique role of the PCZn binder in the SEI formation, XPS was performed with varying Ar^+^ sputtering times on both electrodes after 50 cycles. As shown in Fig. 6a and b, negligible Si 2p signals were obtained for both electrodes before Ar^+^ sputtering. After 24 s of sputtering, two peaks assigned to Li_4_SiO_4_ and Li_*x*_Si were obtained for the Si–PCZn electrode.^42^ However, the spectrum for the Si–PAA electrode remained featureless, indicating the formation of a thicker SEI. Following 48 s of sputtering, Li_*x*_Si-related peaks emerged in the spectrum of the Si–PAA electrode; in the Si–PCZn spectrum, the Li_*x*_Si peak intensity increased, indicating proximity to active Si surfaces. After 72 s of sputtering, a distinct Si peak appeared in the Si–PCZn electrode spectrum but not in that for the Si–PAA electrode. This result indicates that the Si–PCZn electrode exhibited higher electrochemical lithiation–delithiation reversibility.

Fig. 6c and d show F 1s spectra with peaks at 684.9 (LiF) and 686.7 eV (Li_*x*_PF_*y*_).^42^ Throughout the sputtering period of 0–72 s, the LiF content of the Si–PCZn electrode consistently exceeded that of the Si–PAA, confirming LiF-enriched SEI formation. The C 1s spectra (Fig. S23) revealed characteristic peaks at 288.8 (Li_2_CO_3_), 287 (C=O), 286.4 (C–O), and 284.8 eV (C–C).^11^ Progressive intensity reduction of organic signatures (C=O/C–O) with sputtering depth was observed, indicating diminished organic content in the inner SEI regions. These organic peaks were substantially weaker in the Si–PCZn electrode spectrum compared to those in the Si–PAA electrode spectrum, reflecting lower overall organic content, which is consistent with the weaker EC reduction peak in the CV curve. Additionally, the O 1s spectra (Fig. S24) exhibited peaks at 532.4 (C–O) and 531.25 eV (C=O) before sputtering. After 24 s of sputtering, a peak emerged at 528.7 eV (Li_2_O).^42^ During the sputtering period of 24–72 s, the Li_2_O intensity remained higher for the Si–PCZn electrode than for the Si–PAA electrode, signifying enhanced Li_2_O content that benefited the Si particle integrity. Collectively, these results confirm that PCZn promotes a thin, robust LiF-rich SEI on Si surfaces. To visualize SEI stability, TEM and EDS imaging were performed on Si particles after 50 charge–discharge cycles (Fig. 6e and f). TEM showed structurally intact Si particles in Si–PCZn, with EDS-confirmed homogeneous Si and F distributions across the particle surface, indicating uniform LiF-rich SEI coverage. In contrast, particle fracture with thick, disordered SEI encapsulation was observed for Si–PAA. Both XPS depth profiling and TEM demonstrated the efficacy of PCZn in suppressing repetitive SEI growth and maintaining structural stability.

Theoretical calculations were performed to elucidate the mechanistic origin of the LiF-rich SEI formation enabled by the PCZn binder. Molecular electrostatic potential mapping of ZnGa, PF_6_^−^, and FEC (Fig. 6g) revealed complementary polarization: negative electrostatic potentials (electron-rich regions) were obtained for the PF_6_^−^ anions and FEC molecules, whereas positive potentials (electron-deficient sites) were obtained for ZnGa. This charge distribution suggests attraction between ZnGa and PF_6_^−^, along with electrostatic interactions with the polar groups of FEC (carbonyl oxygen); thus, a theoretical basis for their favorable adsorption at the electrode interface was established.^43^

Computational analysis of the charge density differences revealed the charge-transfer process among COS, FEC, and LiPF_6_. During the decomposition of LiPF_6_ and FEC, both COS and PAA donated electrons to the decomposing species, with COS exhibiting a significantly higher charge-transfer value than PAA. These results demonstrate that COS facilitates the cleavage of C–F and P–F bonds by promoting electron transfer toward reactive sites and enhancing charge migration to the reaction zones (Fig. 6h and S25).^44^ The results of these theoretical calculations demonstrate that the synergistic effect between the ZnGa and COS in the PCZn binder effectively promotes the formation of an LiF-rich SEI. This finding agrees closely with the shift and increased intensity of the FEC decomposition peak in the CV test.

Scanning electron microscopy (SEM) was employed to examine the structural integrity of each of the Si electrodes with different binders after 50 and 200 cycles. As shown in Fig. S26, the Si–PCZn electrode exhibited only a moderate thickness increase of 22% was observed. In contrast, the Si–PAA electrode exhibited a substantial over 60% expansion. This result demonstrates the superior ability of the PCZn binder to buffer Si volume expansion. Top-view SEM images of the electrodes before and after cycling are shown in Fig. S27. The cycled Si–PCZn electrode maintained a dense, smooth surface with only minor microcracks, and the overall structural integrity was preserved. By contrast, severe cracking, surface roughening, and indications of active-material delamination were observed for the Si–PAA electrode, reflecting significant volume expansion and structural degradation. Complementary AFM measurements further confirmed the structural stability (Fig. S28 and S29). A modest increase in surface roughness from 73.3 (pristine) to 95.6 nm (after 50 cycles) was observed for the Si–PCZn electrode. In contrast, for the Si–PAA electrode, a drastic roughness increase from 103 to 168 nm was observed, which was consistent with severe surface cracking. Collectively, both the SEM and AFM analyses demonstrated that the PCZn binder effectively alleviates volume expansion and mitigates stress concentration during the Si lithiation–delithiation processes.

## Experimental

### Materials

Polyacrylic acid (PAA; MW ≈ 450 000) was purchased from Sigma Co., Ltd Si powder (≈190 nm) was purchased from Adamas Co., Ltd Chitosan oligosaccharide (COS; MW ≤ 1000) and zinc gluconate (ZnGa) were purchased from Mackin Co., Ltd Super P powder was purchased from Kjsri Co., Ltd All reagents were used without further purification.

### Synthesis of PCZn

First, PAA, ZnGa, and COS were prepared into uniform aqueous solutions with mass ratios of 15%, 5%, and 5%, respectively. The PAA solution was sonicated for 5 min. Then, COS and ZnGa solutions were added to make the mass ratio of PAA : COS : ZnGa equal 90 : 5 : 5. The mixture was stirred at room temperature overnight to ensure that all components reacted fully. The PCZn binder was obtained by diluting the solution to 5%.

### Preparation of electrodes

The slurry was prepared by mixing silicon powder (Fig. S30 and S31) (70 wt%), super P (15 wt%), and binder (PCZn or PAA) (15 wt%). It was then spread on a copper foil and dried in a vacuum oven for 12 h at 80 °C. Regarding the electrode loading, (Si anode: 1 mg cm^−2^, corresponding to 1.94 mAh cm^−2^; NCM811 cathode: 1.84 mAh cm^−2^). Lithium metal foil served as the reference anode; a polypropylene separator was used as the cell separator.

### Cell assembly

The performance of the working electrode was evaluated by assembling CR2032 coin cells. Lithium hexafluorophosphate (LiPF_6_; 1.0 M) in the solvent composed of EC, diethylene carbonate (DEC), and ethyl methyl carbonate (DMC) (1 : 1 : 1, v/v/v) was used as the electrolyte with 5 wt% FEC and 5 wt% VC additives, and was purchased from DodoChem Corporation. For each electrode, 80 µL of electrolyte was used. To assemble full cells, pristine LiNi_0.8_Co_0.1_Mn_0.1_O_2_ (NCM811) electrodes were used as the cathode, and the precycled Si–PCZn and Si–PAA (3 cycles at 0.1C) electrodes were used as the anode. The NCM811 cathode was fabricated by preparing a slurry consisting of NCM811, Super P, and PVDF in a weight ratio of 90 : 5 : 5 in NMP. The slurry was cast onto aluminum foil and dried under vacuum. The N/P ratio, defined by the areal capacity ratio between the anode and cathode, was 1.05 : 1.

### Electrochemical test

To evaluate the electrochemical performance of coin-type half cells, galvanostatic charge–discharge cycling tests were performed using a Neware battery testing system (China). The EIS were obtained at frequencies ranging from 100 kHz to 0.1 Hz. CV was performed at a scan rate of 0.1 mV on a CHI600E electrochemical workstation. To conduct *ex situ* EIS measurement, the battery was discharged or charged to a specific voltage and then tested at an electrochemical workstation.

### Characterization of materials

The phase characterization was performed by X-ray diffraction (XRD; Bruker D8 Advance) at 2*θ* angles between 10° and 80° with Cu Kα radiation (*λ* = 1.5406 Å). Raman spectra were measured with a confocal Raman spectrometer (HR evolution) using a 633-nm laser. The elemental composition and valence state of the materials were characterized by XPS (Escalab 250Xi) using monochromatic Al K*α* radiation with a scanning range from 1 to 1500 eV. The powder and electrode were directly stuck onto the SEM specimen stage using conductive tape. The sizes of the samples were collected from low-magnification SEM (SU8010, Hitachi) images and analyzed by Nano Measure software. FTIR spectra were recorded on a Nicolet iS50 FTIR spectrometer from Thermo Scientific. The rheology experiment was performed at 30 °C using a rotary rheometer (Kinexus Pro) to identify the shear viscosities of the binder solutions (2 wt%) at different shear rates. For the 180° peel test, the electrode sample was attached to 3 M tape based on a glass slip. Both ends of the samples were loaded on a universal testing machine (Zwick/Roell Z020), and the 3 M tape was pulled at a constant displacement rate while the peeling force was monitored. Peak-force quantitative nanomechanical mapping AFM (Dimension icon, Bruker) experiments were performed to measure the adhesion, Young's modulus, and energy dissipation. The morphology and structure of the samples were characterized using TEM (Titan Themis Cubed G2 300).

### Computational details

The first-principles calculations were carried out using the Vienna *ab initio* simulation package (VASP).^45,46^ The projector augmented wave (PAW) of generalized gradient approximation (GGA) was employed with Perdew–Burke–Ernzerhof (PBE) formalism.^47,48^ The plane-wave energy cutoff was set as 520 eV. Configurations were allowed to relax until the energy converged to 1.0 × 10^−5^ eV atom^−1^ and force converged to 0.01 eV A^−1^. The 1 × 1 × 1 *k*-point mesh was employed for the geometry optimizations of the configuration. The dipole–dipole process was performed on the Materials Studio software. A long-range dispersion-corrected DFT-D3 method was incorporated to describe the van der Waals interactions. The hydrogen bond energy was calculated using the following equation: *E*_hydrogen_ = EA + B − EA − EB, where EA + B is the total energy of the system, and EA and EB are energies of the sole molecule. The covalent bond energy was calculated using the following equation: *E*_covalent_ = EPAA–COO–A + EH_2_O − (EPAA–COOH + EA), where EPAA–COO–A is the energy of the system, and EH_2_O, EPAA–COOH, and EA are energies of the sole molecule.

## Conclusions

In summary, PCZn, a multifunctional polymeric binder, was designed by exploiting triadic interactions among PAA chains, a COS crosslinker, and a ZnGa cation donor. This synergistic integration formed a triangular architecture that simultaneously provided robust interfacial adhesion, a mechanically elastic framework, locally distributed fluorine-absorbing charges, and enhanced ionic conductivity. Applied to Si electrodes, the PCZn binder imparted three critical advantages: highly reversible strain tolerance, conformal LiF-rich SEI formation, and accelerated ion transport. With these attributes, the PCZn binder enabled the Si electrode to deliver exceptional electrochemical performance, retaining 1210 mAh g^−1^ after 450 cycles at 3 A g^−1^ and achieving a fast-charging capacity of 1468 mAh g^−1^ at 8 A g^−1^. These results demonstrate the effectiveness of this synergistic multifunctional binder design in enhancing LIB performance.

## Author contributions

Zhipeng Wang: conceptualization (equal); data curation (lead); formal analysis (equal); investigation (lead); methodology (equal); project administration (equal); writing—original draft (equal). Qitao Shi: conceptualization (equal); data curation (equal); formal analysis (equal); supervision (equal); writing—original draft (equal); writing—review and editing (supporting). Weiqi Song: conceptualization (equal); data curation (equal); formal analysis (equal); supervision (equal); writing—original draft (equal); writing—review and editing (supporting). Luwen Li: data curation (supporting); formal analysis (supporting); investigation (supporting); methodology (supporting); writing—original draft (supporting). Jiaqi Wang: data curation (supporting); formal analysis (supporting); investigation (supporting); methodology (supporting). Cheng Zhang: data curation (supporting); formal analysis (supporting); investigation (supporting); methodology (supporting); writing—original draft (supporting). Alicja Bachmatiuk: data curation (supporting); methodology (supporting); resources (supporting); supervision (supporting); writing—review and editing (supporting). Cheng Lu: formal analysis (supporting); investigation (supporting); supervision (supporting); writing—original draft (supporting). Peichao Zou: formal analysis (supporting); investigation (supporting); supervision (supporting); writing—original draft (supporting). Jinho Choi: formal analysis (supporting); investigation (supporting); supervision (supporting); writing—original draft (supporting). Yanbin Shen: formal analysis (supporting); investigation (supporting); supervision (supporting); writing—original draft (supporting). Ruizhi Yang: conceptualization (supporting); data curation (supporting); resources (supporting); writing—original draft (supporting). Mark H. Rümmeli: conceptualization (equal); data curation (equal); formal analysis (equal); funding acquisition (lead); resources (equal); supervision (equal); writing—original draft (lead); writing—review and editing (lead). Zhipeng Wang, Qitao Shi, Weiqi Song contributed equally to this work.

## Conflicts of interest

The authors declare no conflicts of interest.

## Abbreviations

AFM: Atomic force microscopy
CE: Coulombic efficiency
COS: Chitosan oligosaccharide
CV: Cyclic voltammetry
DSC: Differential scanning calorimetry
EC: Ethylene carbonate
EDS: Energy-dispersive spectroscopy
EIS: Electrochemical impedance spectroscopy
GGA: Generalized gradient approximation
GITT: Galvanostatic intermittent titration technique
ICE: Initial Coulombic efficiency
LIB: Lithium-ion battery
PAA: Polyacrylic acid
PAW: Projector augmented wave
SEI: Solid electrolyte interface
SEM: Scanning electron microscopy
TEM: Transmission electron microscopy
XPS: X-ray photoelectron spectroscopy
XRD: X-ray diffraction

## Supplementary Material

SC-OLF-D5SC09750A-s001

## Data Availability

The data that support the findings of this study are available in the article and its supplementary information (SI). Supplementary information: calculated energies, XPS profiles, zeta potential, EIS data, distributions, Raman spectra, Coulomb efficiency, cycling stability, cyclic voltammetry, SEM data, AFM results, XRD characterization, and comparison of specific capacity, cycle numbers, and current density (PDF). See DOI: https://doi.org/10.1039/d5sc09750a.
