# Supplementary material for: A fluorine-absorbing and mechanically elastic binder with triangular architecture enables both bulk- and interface-stable Si anodes
Source: Chem Sci. 2026 Jan 26;17(12):6005–16. doi: 10.1039/d5sc09750a (PMC12856572; doi:10.1039/d5sc09750a)
Supplement: SC-017-D5SC09750A-s001 [file SC-017-D5SC09750A-s001.pdf]

Supplementary Information (SI) for Chemical Science.
This journal is © The Royal Society of Chemistry 2026

Supporting Information

A fluorine-absorbing and mechanically elastic binder with triangular architecture enables both bulk- and interface-stable Si anodes

Zhipeng Wang, Qitao Shi, Weiqi Song, Luwen Li, Jiaqi Wang, Cheng Zhang, Alicja Bachmatiuk, Chen Lu, Peichao Zou, Jinho Choi, Yanbin Shen, Ruizhi Yang, and Mark H. Rümmeli*

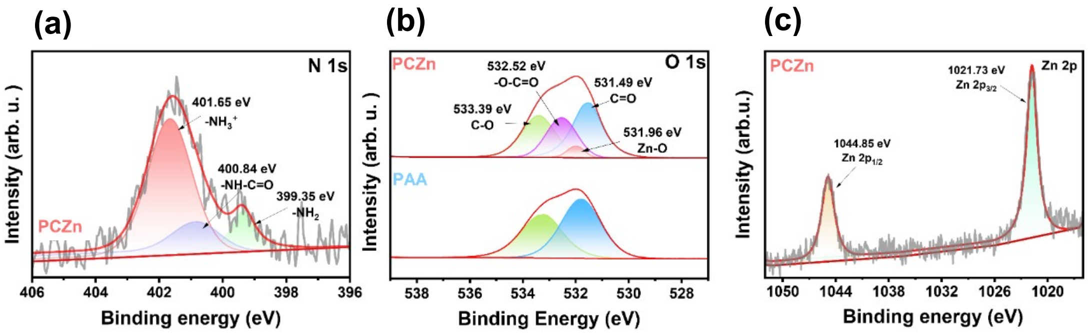

Fig. S1 (a) N 1s, (b) O 1s, and (c) Zn 2p high-resolution XPS spectra of PCZn and PAA.

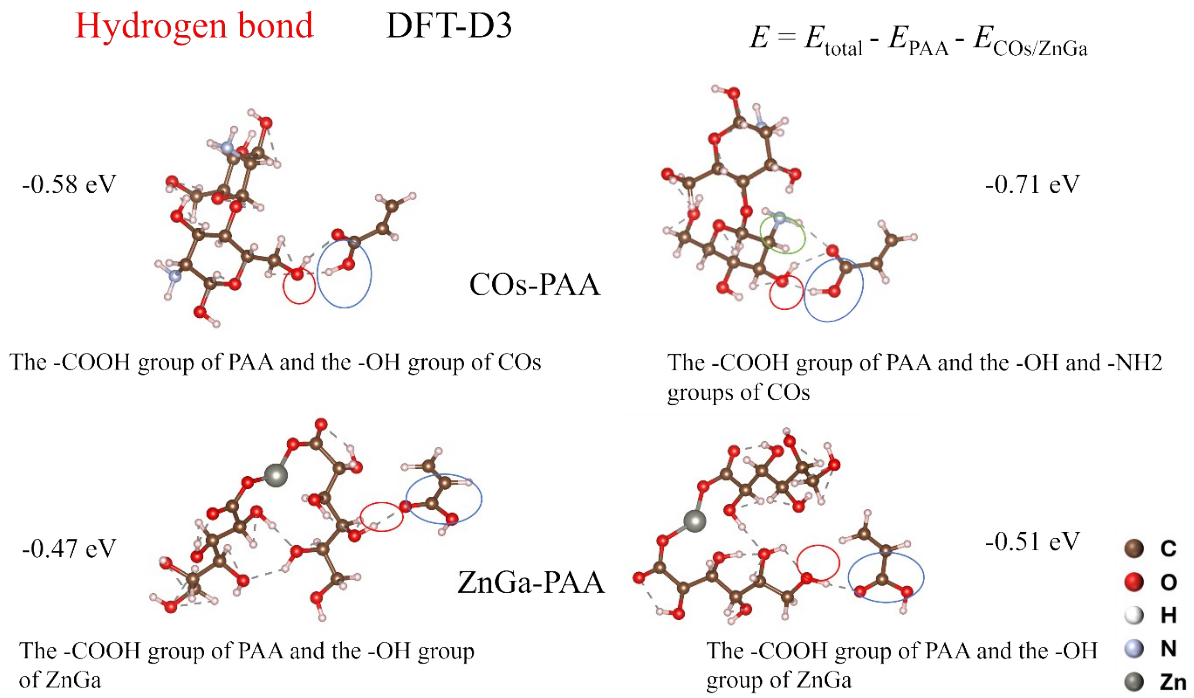

Fig. S2 Calculated energy of gradient hydrogen bonds.

Covalent bond

DFT-D3

COs-PAA

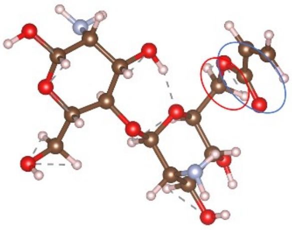

$$E = E_{\text{PAA-COO-COs}} + E_{\text{H2O}} - (E_{\text{PAA}} + E_{\text{COs}})$$

-0.80 eV

-COOH in PAA and -OH in ZnGa combine to form water

ZnGa-PAA

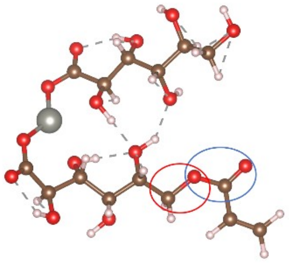

$$E = E_{\text{PAA-COO-ZnGa}} + E_{\text{H2O}} - (E_{\text{PAA}} + E_{\text{ZnGa}})$$

-0.55 eV

The -COOH in PAA combines with the -OH in ZnGa to form water.

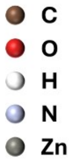

Fig. S3 Calculated energy of covalent bonds.

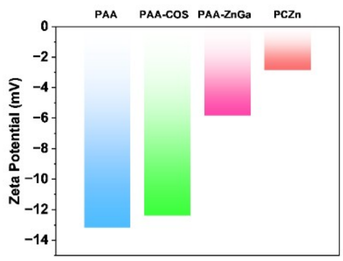

Fig. S4 Zeta potential of PAA, PAA-COS, PAA-ZnGa, and PCZn.

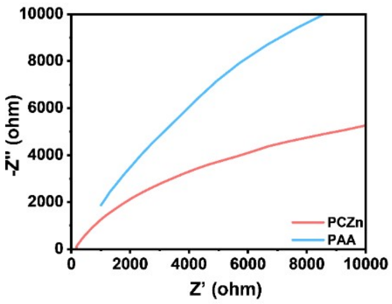

Fig. S5 EIS results for the calculation of ionic conductivity of different binder films.

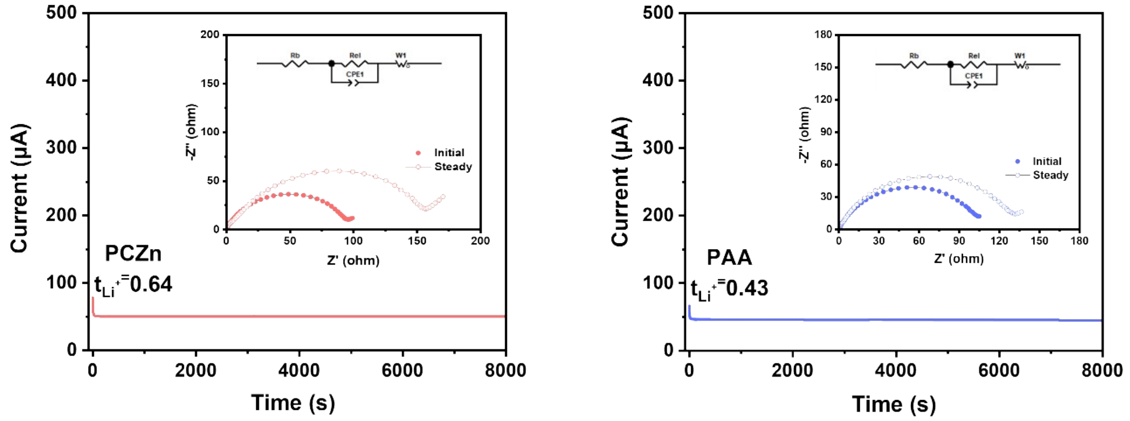

Figure S6. Chronoamperometry profiles of Li|PCZn|Li, Li|PAA|Li, cells. The insets of the figures are Nyquist plots of cells and corresponding equivalent circuits.

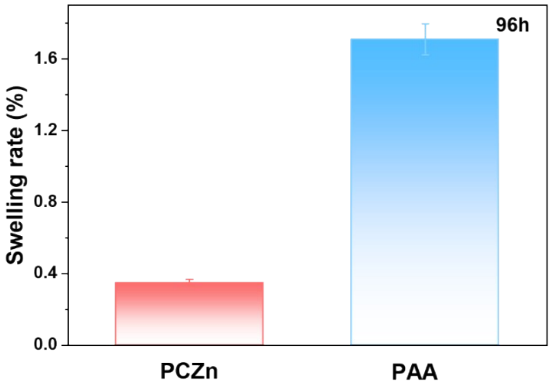

Figure S7. The swelling rate of PCZn and PAA films in the electrolyte

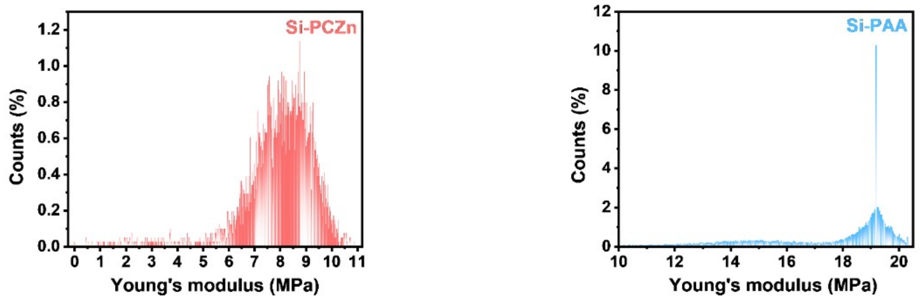

Fig. S8 Young's modulus distribution of Si-PCZn and Si-PAA.

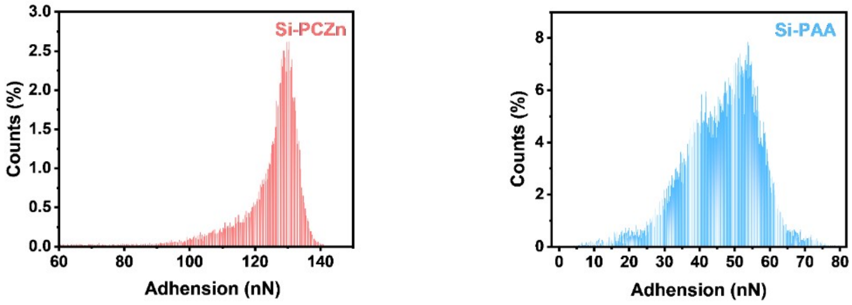

Fig. S9 Adhesion distribution of Si-PCZn and Si-PAA.

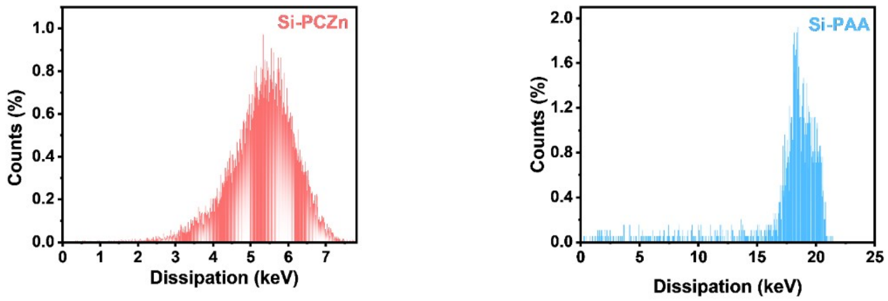

Fig. S10 Dissipation distribution of Si-PCZn and Si-PAA.

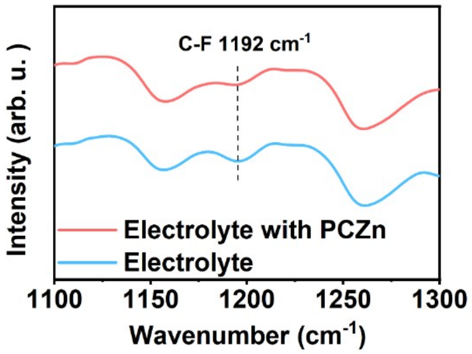

Fig. S11 FTIR spectra of electrolyte and electrolyte with PCZn.

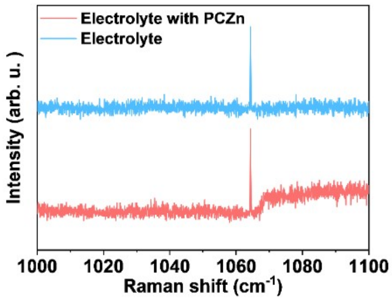

Fig. S12 Raman spectra of the electrolyte and electrolyte with PCZn.

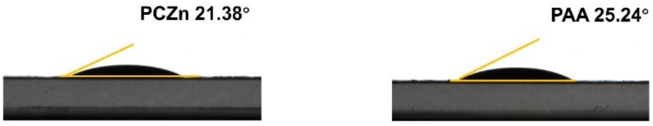

Fig. S13 Contact angles of electrolyte on Si electrodes with PCZn and PAA binders at 0 s.

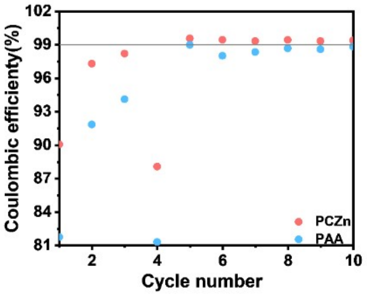

Fig. S14 Coulombic efficiency of Si-PCZn and Si-PAA electrodes within 10 cycles.

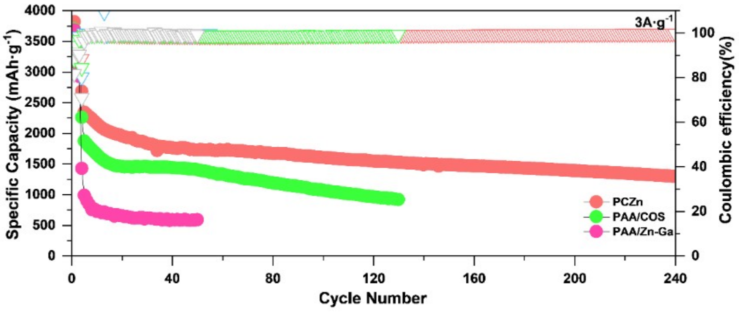

Fig. S15 Long-time cycling stability at 1 C of the Si-PCZn, Si-PAA/COS, and Si-PAA/ZnGa electrodes.

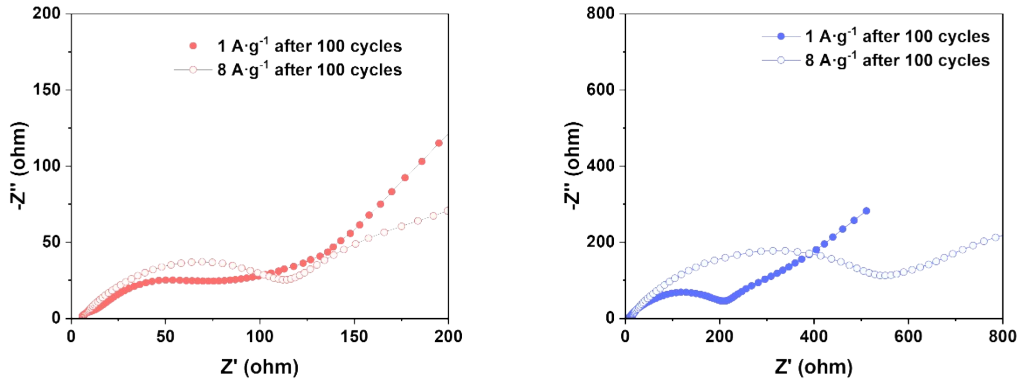

Fig. S16 EIS spectr after 100 cycles at different current densities of Si-PCZn and Si-PAA

EIS fitting data values of Si-PCZn at different current densities

Binder	Different current densities	R_{ct} (Ω)	R_{SEI} (Ω)
PCZN	1 A/g	141	60
	8 A/g	128	71
PAA	1A/g	232	150
	8A/g	598	309

The Si-PCZn electrode demonstrates exceptional interfacial stability. After cycling at 8 A g^{-1}, its charge-transfer resistance (Rct) slightly decreases and its SEI resistance (Rsei) increases only minimally, resulting in a nearly unchanged total interface impedance compared to its state at 1 A g^{-1}. This confirms the formation of a stable, highly conductive interface.

In stark contrast, the Si-PAA electrode suffers severe interfacial degradation at 8 A g^{-1}, with Rsei and Rct increasing by ~106% and ~158%, respectively. The Rct becomes the major impedance component (598 Ω), identifying severely hindered charge-transfer kinetics as the

primary performance bottleneck. The concurrent surge in Rsei further indicates the formation of a thick, resistive SEI under high-rate stress.

Thus, at ultra-high current densities, the primary limitation for conventional binders is drastic charge-transfer kinetic degradation, exacerbated by an unstable SEI. Our Si-PCZn binder effectively mitigates both issues.

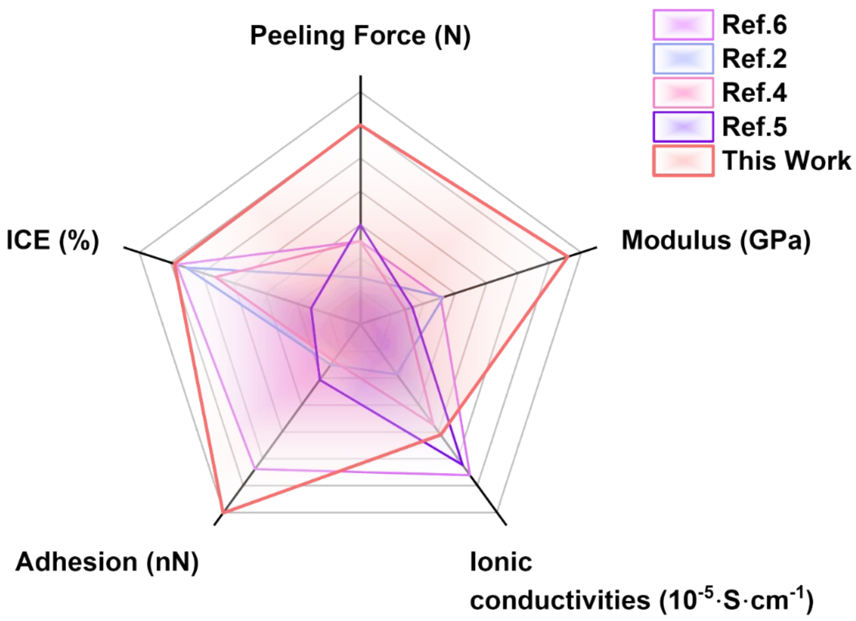

Fig. S17. Radar chart of ICE, Adhesive strength, Modulus, Peeling Force and Ionic conductiviyies of different binders.

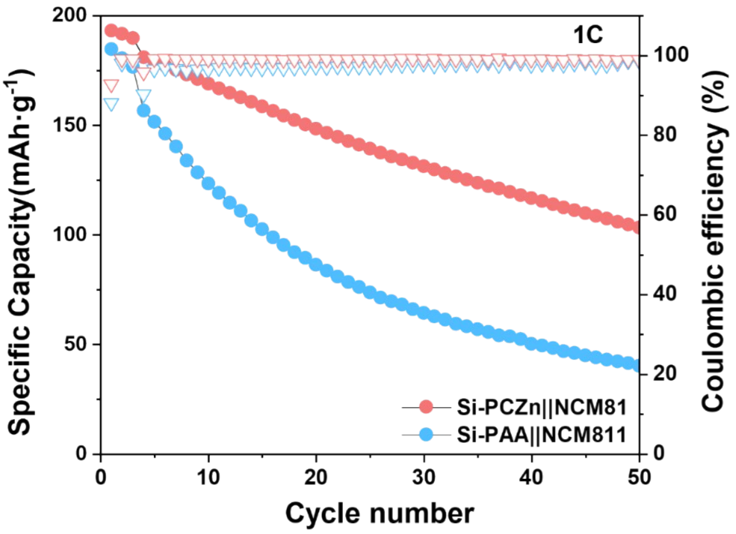

Fig. S18 Cycling performance of Si-PCZn||NCM811 and Si-PAA||NCM811full cells at 1C.

.

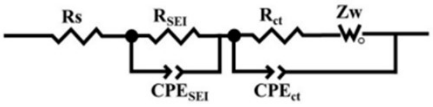

Fig. S19 Equivalent circuit used for fitting the EIS spectra.

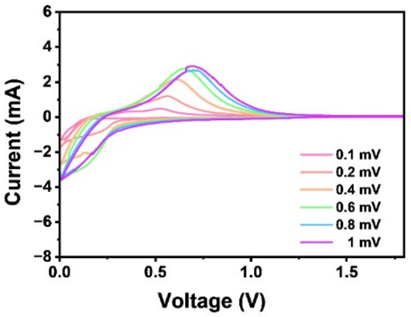

Fig. S20 CV curves of Si-PAA at different sweep rates from 0.1 to 1 $mV \cdot s^{-1}$.

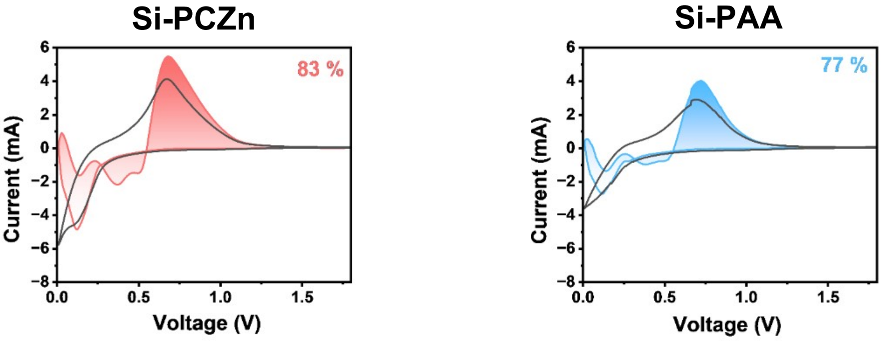

Fig. S21. Separation of the capacitive and diffusion currents in the Si-PCZn and Si-PAA electrodes at 1.0 mV s^{-1}.

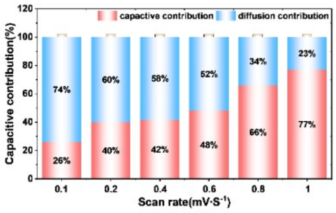

Fig. S22 Capacitance contribution of the Si-PAA electrode at various sweep rates.

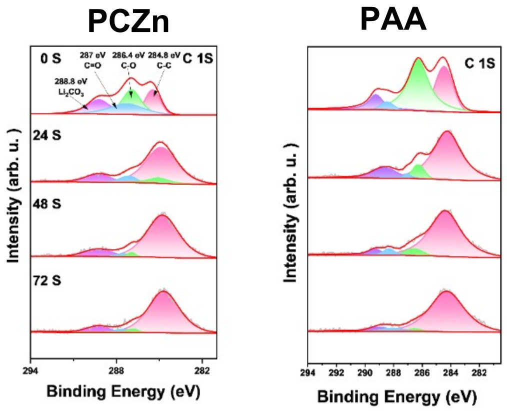

Fig. S23. High-resolution XPS spectra of C 1s from the cycled Si electrodes.

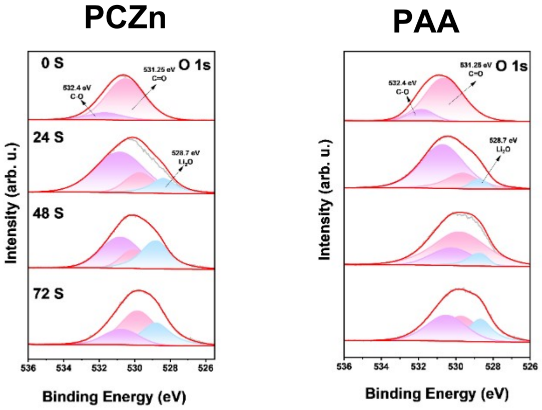

Fig. S24 High-resolution XPS spectra of O 1s from the cycled Si electrodes.

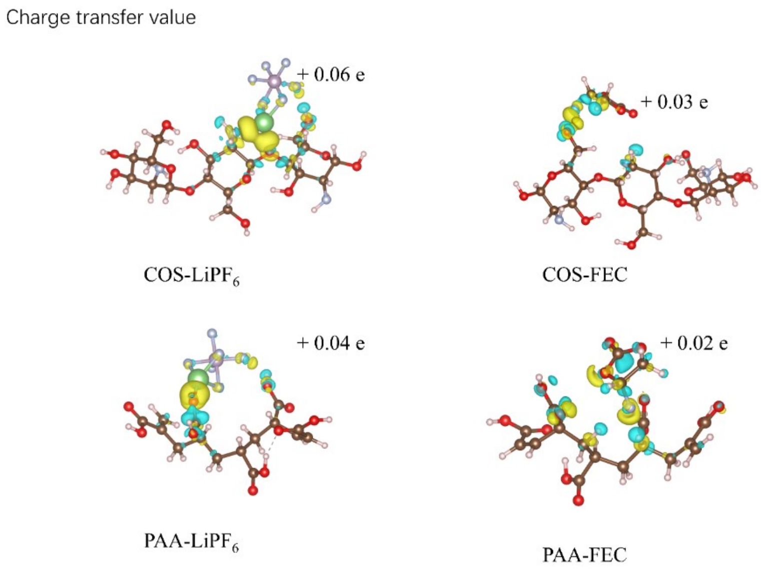

Fig. S25 Charge density difference of PAA and COS**.**

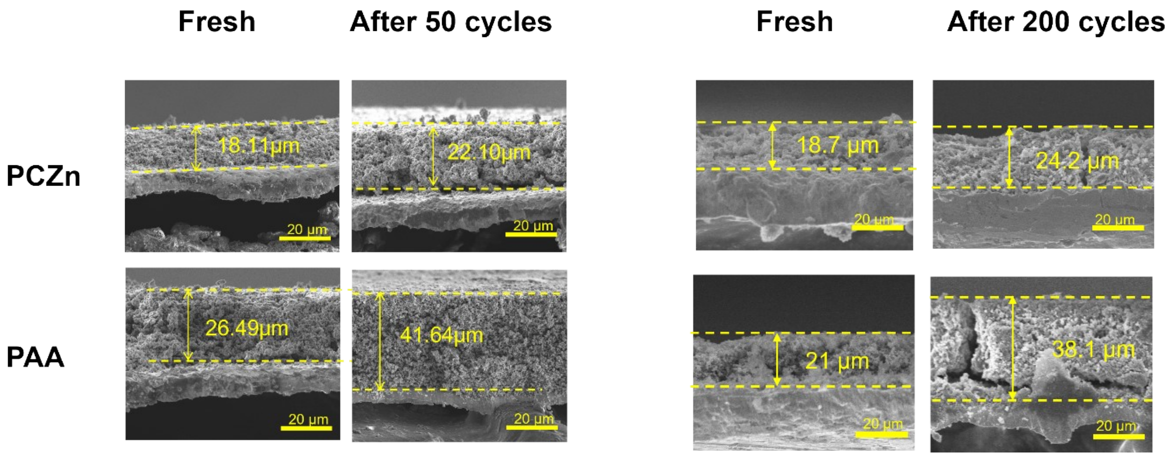

Fig. S26 Cross-section SEM images of Si-PCZn and Si-PAA electrodes.

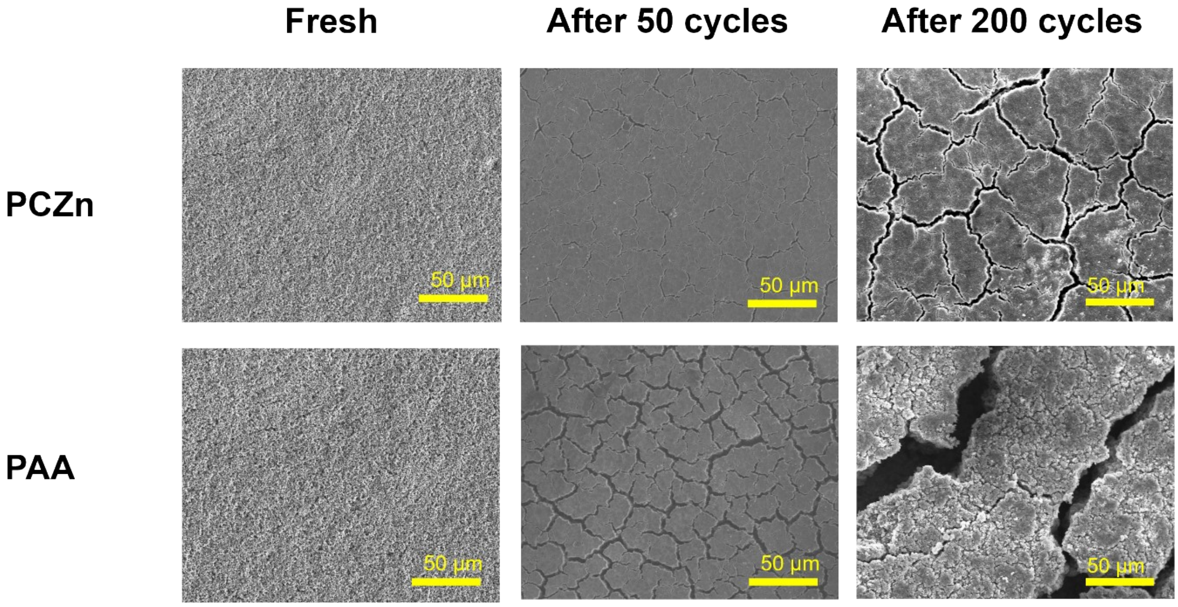

Fig. S27 Surface SEM images of Si-PCZn and Si-PAA electrodes.

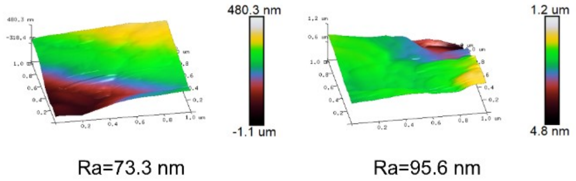

Fig. S28AFM images of the Si-PCZn electrodes before and after 50 cycles.

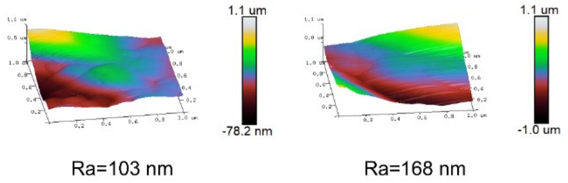

Fig. S29 AFM images of the Si-PAA electrodes before and after 50 cycles.

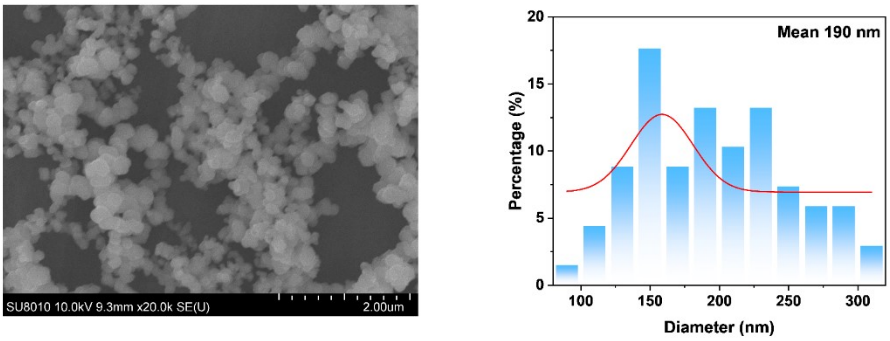

Fig. S30 SEM image and size distribution of SiNPs.

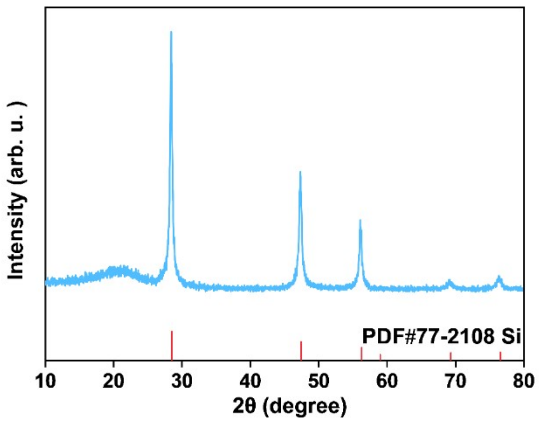

Fig. S31 XRD patterns of SiNPs.

Table S1. Comparison of specific capacity, cycle numbers, and current density of the Si-PCZn electrode with previously reported Si-based anodes.

Si-based anodes	Specific capacity ($mAh\cdot g^{-1}$)	Cycle numbers	Current density ($A\cdot g^{-1}$)	Reference
PCZn	1210	450	3	**This work**
TA-c-PAA	1742	450	1	1
PTBR	1968	200	2	2
CSS	1600	240	0.8	3
GG-g-PAM	750	500	2	4
Li_xPAA	922	400	0.5	5
GG-g-PAA	2137	220	2.1	6
PAA-TA	1025	250	0.5	7
CGG	1138	200	1	8

Table S2. EIS fitting data values of different binders after different cycles

**Binder**	Different cycles	R_{ct} (Ω)	R_{SEI} (Ω)
**PCZN**	Fresh	12	16
	10th	127	79
	20th	147	90
	50th	190	104
	100th	227	106
**PAA**	Fresh	15	20
	10th	140	94
	20th	164	87
	50th	229	148
	100th	432	260

References

1 J. Chen, Y. Li, X. Wu, H. Min, J. Wang, X. Liu and H. Yang, *J. Colloid Interface Sci.*, 2024, **657**, 893–902.

2 B. Zhang, Y. Dong, J. Han, Y. Zhen, C. Hu and D. Liu, *Adv. Mater.*, 2023, **35**, e2301320.

3 K. Hu, J. Chen, J. Zhang, X. Sang, T. Meng, Z. Wang and X. Hu, *Energy Storage Mater.*, 2025, **75**, 104029.

4 Z. Li, G. Wu, Y. Yang, Z. Wan, X. Zeng, L. Yan, S. Wu, M. Ling, C. Liang, K. N. Hui and Z. Lin, *Adv. Energy Mater.*, 2022, **12**, 2201197.

5 Z. Li, W. Tang, Y. Yang, G. Lai, Z. Lin, H. Xiao, J. Qiu, X. Wei, S. Wu and Z. Lin, *Adv. Funct. Mater.*, 2022, **32**, 2206615.

6 Z. Li, Z. Wan, Z. Lin, M. Zheng, J. Zheng, S. Qian, Y. Wang, T. Song, Z. Lin, and J. Lu, *Energy Environ. Sci.*, 2025, **18**, 2365–2380.

7 W. Tang, L. Feng, X. Wei, G. Lai, H. Chen, Z. Li, X. Huang, S. Wu and Z. Lin, *ACS Appl. Mater. Interfaces*, 2022, **14**, 56910–56918.

8 S. Hu, Z. Cai, T. Huang, H. Zhang and A. Yu, *ACS Appl. Mater. Interfaces*, 2019, **11**, 4311–4317.
